# Light Regimes Shape Utilization of Extracellular Organic C and N in a Cyanobacterial Biofilm

**DOI:** 10.1128/mBio.00650-16

**Published:** 2016-06-28

**Authors:** Rhona K. Stuart, Xavier Mayali, Amy A. Boaro, Adam Zemla, R. Craig Everroad, Daniel Nilson, Peter K. Weber, Mary Lipton, Brad M. Bebout, Jennifer Pett-Ridge, Michael P. Thelen

**Affiliations:** a Lawrence Livermore National Laboratory, Livermore, California, USA; b Pacific Northwest National Laboratory, Richland, Washington, USA; c Exobiology Branch, NASA Ames Research Center, Moffett Field, California, USA

## Abstract

Although it is becoming clear that many microbial primary producers can also play a role as organic consumers, we know very little about the metabolic regulation of photoautotroph organic matter consumption. Cyanobacteria in phototrophic biofilms can reuse extracellular organic carbon, but the metabolic drivers of extracellular processes are surprisingly complex. We investigated the metabolic foundations of organic matter reuse by comparing exoproteome composition and incorporation of ^13^C-labeled and ^15^N-labeled cyanobacterial extracellular organic matter (EOM) in a unicyanobacterial biofilm incubated using different light regimes. In the light and the dark, cyanobacterial direct organic C assimilation accounted for 32% and 43%, respectively, of all organic C assimilation in the community. Under photosynthesis conditions, we measured increased excretion of extracellular polymeric substances (EPS) and proteins involved in micronutrient transport, suggesting that requirements for micronutrients may drive EOM assimilation during daylight hours. This interpretation was supported by photosynthesis inhibition experiments, in which cyanobacteria incorporated N-rich EOM-derived material. In contrast, under dark, C-starved conditions, cyanobacteria incorporated C-rich EOM-derived organic matter, decreased excretion of EPS, and showed an increased abundance of degradative exoproteins, demonstrating the use of the extracellular domain for C storage. Sequence-structure modeling of one of these exoproteins predicted a specific hydrolytic activity that was subsequently detected, confirming increased EOM degradation in the dark. Associated heterotrophic bacteria increased in abundance and upregulated transport proteins under dark relative to light conditions. Taken together, our results indicate that biofilm cyanobacteria are successful competitors for organic C and N and that cyanobacterial nutrient and energy requirements control the use of EOM.

## IMPORTANCE

Cyanobacteria are globally distributed primary producers, and the fate of their fixed C influences microbial biogeochemical cycling. This fate is complicated by cyanobacterial degradation and assimilation of organic matter, but because cyanobacteria are assumed to be poor competitors for organic matter consumption, regulation of this process is not well tested. In mats and biofilms, this is especially relevant because cyanobacteria produce an extensive organic extracellular matrix, providing the community with a rich source of nutrients. Light is a well-known regulator of cyanobacterial metabolism, so we characterized the effects of light availability on the incorporation of organic matter. Using stable isotope tracing at the single-cell level, we quantified photoautotroph assimilation under different metabolic conditions and integrated the results with proteomics to elucidate metabolic status. We found that cyanobacteria effectively compete for organic matter in the light and the dark and that nutrient requirements and community interactions contribute to cycling of extracellular organic matter.

## INTRODUCTION

The extracellular milieu is the interface at which cyanobacteria interact both with their environment and with one another (1), and yet there is much that we do not understand about how these primary producers use this space. Because extracellular composition and activities dictate community resource partitioning and ultimately link to microbial community function, it is important to investigate this extracellular pool of resources (2).

The metabolic foundations behind extracellular processes such as excretion, degradation, and reincorporation are surprisingly complex, particularly for cyanobacteria. Relatively few physiological mechanisms controlling excretions by “leaky” primary producers have been described, although they are thought to account for 25% of primary production in some marine systems (3). For example, excretion of osmolytes (4) (as a response to stress) and excretion of fermentation by-products (5) are well established, but the functional roles of other exopolymers are less clear (6). *Cyanobacteria* such as *Crocosphaera* and those dwelling in microbial mats may use exopolymers for oxygen or UV protection (7, 8); others appear to produce exopolysaccharides to remedy carbon-nitrogen imbalances (9). Microbial interactions also likely play a role in cyanobacterial excretions; for example, marine *Synechococcus* spp. secrete allelopathic chemicals to mediate competition (10).

Cyanobacterial extracellular degradation of organic compounds also plays a role in nutrient cycling. For example, in response to phosphate stress, some marine cyanobacteria secrete alkaline phosphatases to acquire phosphate from organic sources (11, 12). Extracellular nucleases are also common in heterocystous cyanobacterial strains (13). However, extracellular degradation may be the least understood of these processes; genomics-enabled research has revealed a large number of extracellular proteins (exoproteins) of unknown function, implying that a number of extracellular activities remain uncharacterized (14).

Although primarily photoautotrophs, many cyanobacteria also incorporate organic C and N, ranging from phosphonates (15) and amino acids (16, 17) to glucose (18, 19). Light availability is likely to play a role in this incorporation and has been shown to influence the uptake of an important organic sulfur and carbon compound, dimethylsulfoniopropionate, by marine picocyanobacteria and heterotrophs (20). Interestingly, leucine uptake in these coastal picocyanobacterial assemblages is not light-driven (20), but picocyanobacterial amino acid uptake in oligotrophic environments is stimulated by light (21), and the nature of the metabolic regulation behind this light stimulation is unclear (22).

Cyanobacterial biofilms and phototrophic mats provide a relevant and tractable system to study metabolic drivers of photoautotroph organic matter incorporation in the presence of heterotrophs. These systems often contain copious amounts of extracellular polymeric substances (EPS), mainly polysaccharides, proteins, and nucleic acids (23), to which metabolites and inorganic micronutrients may be adsorbed, creating an extracellular pool of resources that is large relative to the size of the active biomass. Members of a single group of organisms, the cyanobacteria, are often the dominant primary producers in phototrophic mats (e.g., 24, and the turnover of organic matter can be rapid (25), suggesting active processes that can be measured to identify contributors to turnover under various conditions.

In a previous study, we characterized extracellular organic matter (referred to here as “EOM”) from a natural microbial mat collected at Elkhorn Slough in Monterey Bay, CA, and a unicyanobacterial biofilm (“cultured biofilm”) (26). The biofilm is comprised of a marine filamentous cyanobacterium, strain ESFC-1, and associated microbes, isolated from the Elkhorn Slough microbial mat into culture. ESFC-1 belongs to a recently identified lineage of active diazotrophs in these mats and can comprise up to 10% of the natural mat cyanobacterial community (27). The biofilms produce exopolysaccharides predominantly composed of either α- or β(1,4)-linked glucans, as does the intact mat (26). Similarly, a number of homologous exoproteins, such as Zn-dependent peptidases and redox-active proteins, are produced by the both the biofilm and the intact mat (26). Accordingly, these biofilms represent a suitable simplified system to study cyanobacterial extracellular processes. Furthermore, in both intact mat and cultured biofilms, enzymes are produced by cyanobacteria that likely function in the degradation of EOM, and ESFC-1 incorporates its own extracellular organic C (26). This led us to hypothesize that the cyanobacteria use the extracellular matrix as a storage reservoir for C and regulate both excretion and assimilation processes.

Here, we used this simplified biofilm system to examine the metabolic and molecular underpinnings of cyanobacterial EOM reuse in cultured biofilms. To test whether the extracellular matrix is used as a storage reservoir, we compared EOM incorporation, composition, and activity using four light regimes: normal diel light-dark treatment, continuous light (C-replete; dissolved inorganic C [DIC] achieved through photosynthesis) treatment, continuous dark (C-starved) treatment, and diel DCMU [3-(3,4-dichlorophenyl)-1,1-dimethylurea] (oxygenic photosynthesis inhibition) treatment. We also investigated N incorporation from EOM, since little is known about the dynamics of organic C/N incorporation by photoautotrophs in aquatic ecosystems. Applying stable isotope tracing at the single-cell level, using the high-resolution imaging secondary ion mass spectrometry (NanoSIMS) method, we integrated these results with proteomics data to provide a more comprehensive picture of the flow of essential nutrients to different community members.

## RESULTS

### Uptake of EOM under various light regimes.

**FIG 1:**
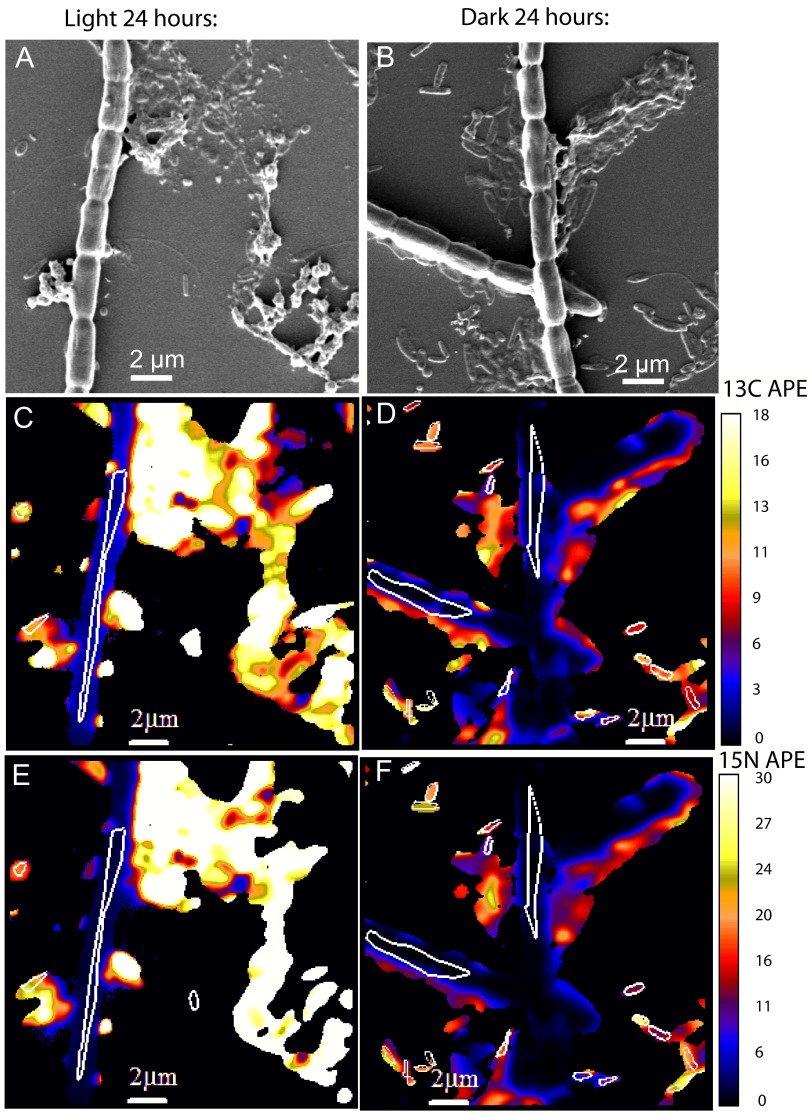
Representative NanoSIMS images of cyanobacterium ESFC-1 trichomes and heterotrophs 24 h after addition of ^13^C-labeled and ^15^N-labeled EOM. Trichomes and associated microbes were identified morphologically by scanning electron microscopy (SEM) prior to NanoSIMS analysis. Left, images (panels A and C and panel E) are paired images from a light-treated biofilm; right, images (panels B and D and panel F) are paired images from a dark-treated biofilm. (A and B) Scanning electron microscope images taken prior to NanoSIMS analysis. White outlines in panels C to F correspond to areas analyzed (ROIs), from which values for individual cells are derived. (C and D) ^13^C enrichment images. (E and F) ^15^N enrichment images. APE, atom percent excess.

**FIG 2:**
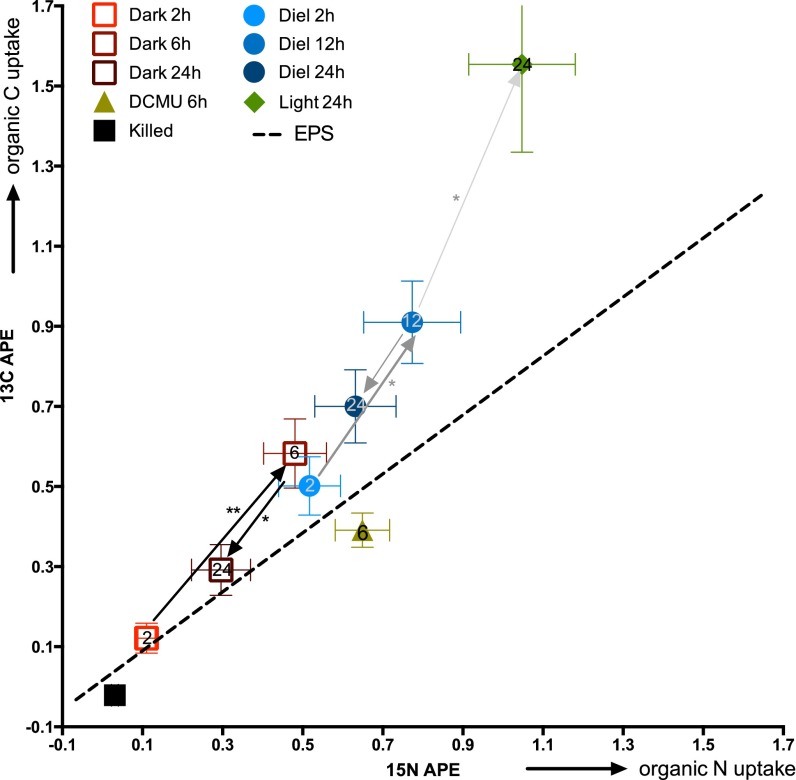
NanoSIMS-derived average levels of ^13^C and ^15^N APE (atom percent excess) of cyanobacterial trichomes for four treatments over time following incubation of unialgal biofilms with ^13^C-labeled and ^15^N-labeled EOM. Error bars represent 1 standard error of the mean. Each point represents an average of at least 14 cells analyzed via NanoSIMS. The dashed line indicates the ^13^C/^15^N ratio of the labeled substrate, as determined by IRMS. *, significant difference between mean ^13^C APE values (*P* < 0.05). **, significant difference between mean ^13^C APE values (*P* < 0.05) and mean ^15^N APE values (*P* < 0.05).

Organic matter additions were examined under three distinct illumination regimes: continuous light, continuous dark, and diel (12-h light/12-h dark) treatments over 24 h. ^13^C-labeled and ^15^N-labeled cyanobacterial EOM was added to unicyanobacterial biofilms at the start of the light phase. The continuous light regime was sampled at the 24-h time point, as data from earlier time points were identical to those in the diel regime. High-resolution imaging secondary ion mass spectrometry (NanoSIMS) was used to quantify ^13^C and ^15^N assimilation in individual cyanobacterial trichomes and in associated microbes (referred to here as “heterotrophs”) (Fig. 1; see also Table S1 in the supplemental material). By 24 h, cyanobacterial C incorporation was higher in the continuous light treatment than in the continuous dark treatment (Fig. 1). To quantify these differences, we calculated mean ^13^C and ^15^N enrichment values for each treatment over time (Fig. 2). On the basis of bulk isotope ratio mass spectrometry (IRMS) analysis, the C/N ratio of the added labeled EOM was 4.8, representing an isotopic enrichment of 67.6 ^13^C atom percent excess (APE) and 85.4 ^15^N APE. The EOM (^13^C APE)/(^15^N APE) ratio was used to establish the line indicating the proportions of C and N uptake from the added EOM (Fig. 2, dashed line) to calculate the C/N incorporated by individual cells.

Under all experimental regimes, cyanobacterial cells incorporated EOM-derived C and N, as shown in Fig. 2. Mean ^13^C and ^15^N APE values were significantly higher than the killed control mean values (*P* < 0.05), except for the 2-h and 24-h dark treatments. When photosynthetic electron transfer was inhibited by DCMU [3-(3,4-dichlorophenyl)-1,1-dimethylurea], ^13^C and ^15^N incorporation at 6-h was still significantly higher than that seen with killed controls (*P* < 0.0001). In the continuous dark treatment, mean cyanobacterial ^13^C incorporation increased, with the maximum measured at 6 h (0.54 ^13^C APE; 0.46 ^15^N APE), and then decreased by ~50% at 24 h (Fig. 2). In the biofilms incubated with a diel light cycle, ^13^C incorporation increased significantly (0.5 to 0.9 APE) between 2 hours and 12 hours of light and then decreased to 0.7 APE following the 12-h dark period, although this was not statistically significant. In the continuous light treatment, mean ^13^C incorporation increased between 12 h and 24 h of light (*P* = 0.0027).

**FIG 3:**
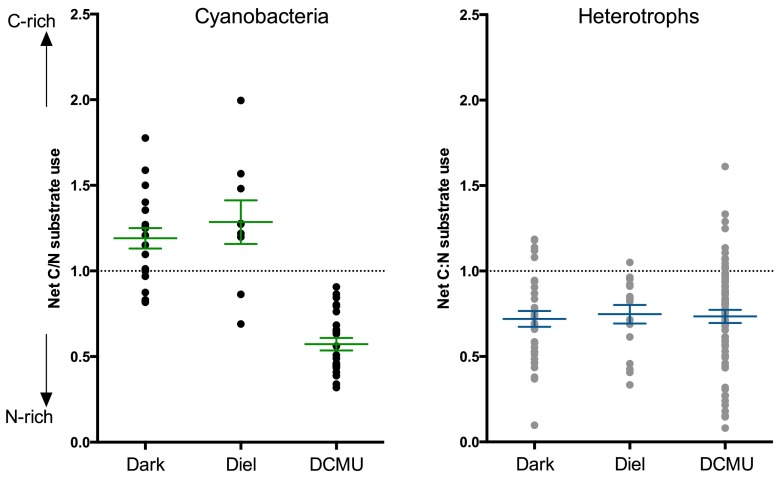
C/N relative assimilation from the substrate, accounting for C/N of both substrate and biomass, based on mass balance calculations. Each point represents calculated C/N substrate usage for an individual cell. (Left panel) Cyanobacteria following 6-h incubations using dark (*n* = 19), diel (*n* = 9), or DCMU (*n* = 24) treatments. (Right panel) Heterotrophs following 12-h dark (*n* = 32) and diel (*n* = 17) treatments and 6-h DCMU (*n* = 70) treatment. Error bars indicate 1 standard error of the mean. All means were significantly different from 1.0 (*P* < 0.05).

To assess preferences for C or N assimilation from the substrate, we calculated C/N relative assimilation levels for individual cells, based on the C/N ratio of the organism and the substrate (28), as shown in Fig. 3. A C/N relative assimilation value of 1.0 indicates that C and N were incorporated in equivalent amounts from the substrate. For cyanobacteria, we compared C/N relative assimilation values at the 6-h time point so that we could include the DCMU treatment as well as the continuous dark and diel regimes. Mean C/N relative assimilation values were 1.25 ± 0.1 and 1.29 ± 0.12 at 6 h in the diel and dark regimes, respectively, indicating a significant C preference in assimilation from the substrate. Photosynthesis inhibition with DCMU at 6 h resulted in a cyanobacterial C/N relative assimilation value of 0.57 ± 0.04, indicating that under normal conditions, a fraction of the EOM-derived C was taken up as DIC. This also indicates that chemolithoautotrophy among the noncyanobacterial microbes (referred to as “heterotrophs”) was not occurring, supporting our presumption that the associated microbes in the biofilm were functioning primarily as heterotrophs.

We used parallel incubations with ^13^C bicarbonate to verify the expected utilization patterns of inorganic C and to determine a bulk rate of DIC incorporation. On the basis of bulk IRMS measurements, as expected, the biofilm culture did not incorporate a detectable amount of dissolved inorganic C (DIC) in the dark (^13^C APE, 0.009 ± 0.003) but incorporated high levels in the light (^13^C APE, 4.56 ± 0.27). This result supports our interpretation that a portion of the EOM-derived C assimilation is from mineralized EOM in the light.

While cyanobacteria and heterotrophs both incorporated EOM, net levels of C/N assimilation differed between these two groups (see Fig. S1 in the supplemental material). For heterotrophs, we compared C/N relative assimilation levels between diel-, continuous dark-, and DCMU-treated cells. Due to an insufficient number of cells analyzed at the 6-h time point for the dark and diel regimes (see Table S1), we also analyzed the 12-h time point and the DCMU treatment at 6 h. Average heterotroph C/N relative assimilation values were 0.72 ± 0.05 and 0.74 ± 0.05 following 12 h in continuous dark and diel, respectively, indicating a significant N preference, regardless of light conditions (Fig. 3). This was significantly different from the C preference displayed by cyanobacteria (Fig. 3).

### Contributions to C and N fluxes in the extracellular reservoir.

To evaluate the effect of light regime on cyanobacterial versus heterotroph C and N uptake, we calculated the average levels of C and N incorporated from the added EOM after 6 h of light, dark, or DCMU treatment conditions and compared the results to those seen with DIC incorporation, measured via bulk IRMS, after 6 h in the light (Table 1). Associated heterotroph cells represented on average 4.2% (±1.36%) of the biomass C, with cyanobacteria accounting for the remaining 95.8%. Since the biovolumes of the replicates were not significantly different over time, we extrapolated the data to calculate the average amounts of C and N (in micrograms) incorporated per well area (in cubic micrometers) (see Materials and Methods). The net fixation of DIC (percent C incorporated relative to the initial DIC pool) was 5.6 µg C incorporated per biofilm. In the light treatment, cyanobacteria incorporated approximately 1.3 µg of C derived from EOM (Table 1). Inorganic nitrogen uptake was not measured, but on the basis of our measured cyanobacterial C/N ratio of 4.0, we estimate that uptake of an additional 1.5 µg N from nitrate (or ammonium) would be necessary to maintain a constant C/N ratio. Given these assumptions, uptake of EOM-derived C and N may account for up to 18% and 10.5% of total cyanobacterial C and N uptake in the light treatment, respectively. In the dark, applying the same assumptions, organic substrate-derived N could account for 59% of N uptake.

**TABLE 1:** Incorporation of C and N by taxonomic groups based on NanoSIMS-derived population averages at 6 h for the cyanobacteria and 12 h for the heterotrophs^a^

Treatment	µg C incorporated/well (± SE)		µg N incorporated/well (± SE)	
	*Cyanobacteria*	Heterotroph	*Cyanobacteria*	Heterotroph
Dark, extracellular	0.93 (0.19)	1.2 (0.23)	0.13 (0.03)	0.55 (0.08)
Diel, extracellular	1.27 (0.30)	1.3 (0.22)	0.18 (0.04)	0.65 (0.16)
DCMU, extracellular	0.60 (0.10)	1.3 (0.23)	0.18 (0.02)	0.89 (0.22)
Light, bicarbonate	5.66 (0.75)			
Dark, bicarbonate	NS^b^			
Nitrate, light (estimated)			1.5	
Nitrate, dark (estimated)			0.09	

a The number of cells analyzed (*n*) was between 9 and 31.

b NS, not significantly different from result seen with killed control.

Despite low biovolume relative to the cyanobacteria, heterotrophs accounted for approximately 68% of EOM-derived C uptake in the DCMU treatment, with cyanobacteria incorporating the remaining 32%. This difference was more pronounced for N, where heterotrophs accounted for 80% of the EOM N uptake in the DCMU treatment (Table 1).

### The effect of light regime on biofilm composition and enzyme activity.

**FIG 4:**
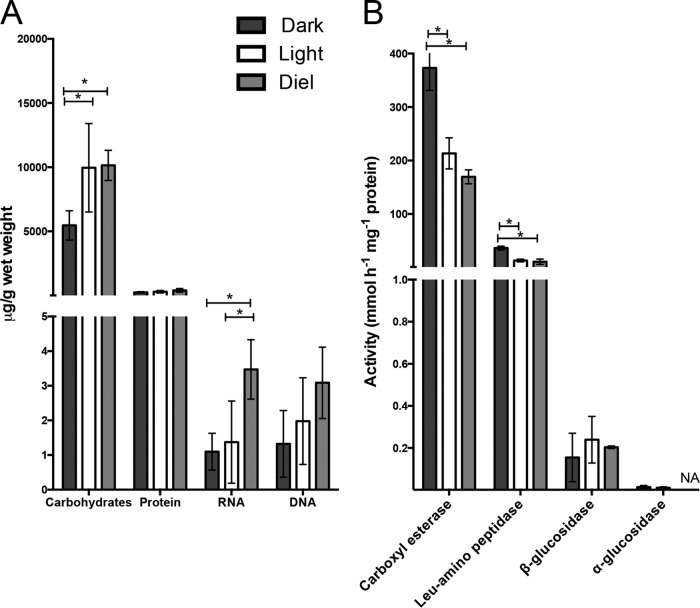
Bulk composition of major EPS polymers and selected enzymatic activities of cyanobacterial biofilm extracellular matrix after 3 days under different light treatments (Dark, 3 days in the dark; Diel, 12-h/12-h light/dark cycle; Light, 3 days in the light. (A) Carbohydrate, protein, and nucleic acids in the extracellular fraction are all normalized to grams of wet weight. Error bars represent 1 standard deviation for results from five biological replicates. *, significant difference between treatments (*P* < 0.05). (B) Four different degradative enzymes were assayed in the extracellular fraction, and for each enzyme, the specific activity was calculated by the rate of degradation of a fluorescent substrate over 3 h. Error bars represent 1 standard error for results from three biological replicates.

After 3 days of treatment using continuous darkness, we measured extracellular composition and enzyme activity in the biofilms and found evidence for both decreased EPS levels and increased degradation activity relative to the continuous light and diel treatments. Measured levels of extracellular carbohydrate and RNA content were significantly lower in the dark treatment (Fig. 4A). Extracellular enzyme assays revealed that protease and carboxyl esterase activities increased significantly in the dark-treated biofilms (Fig. 4B). Additionally, in biofilms kept in continuous darkness, the signal intensity from the exopolysaccharide fluorescent stain, Congo red, was significantly reduced (see Fig. S2 in the supplemental material), corroborating carbohydrate measurements indicating that EPS levels decreased in the dark.

Oxygen levels in the media at the surface of the biofilm were measured after 3 days of incubation and were significantly lower in the dark treatment and higher in the light treatment than in the diel treatment at the 6-h light phase (see Table S2 in the supplemental material). We also measured pH in spent media and found that pH was significantly higher in the light-treated cultures and lower in the dark-treated cultures.

### Cyanobacterial exoprotein expression under light and dark conditions.

**FIG 5:**
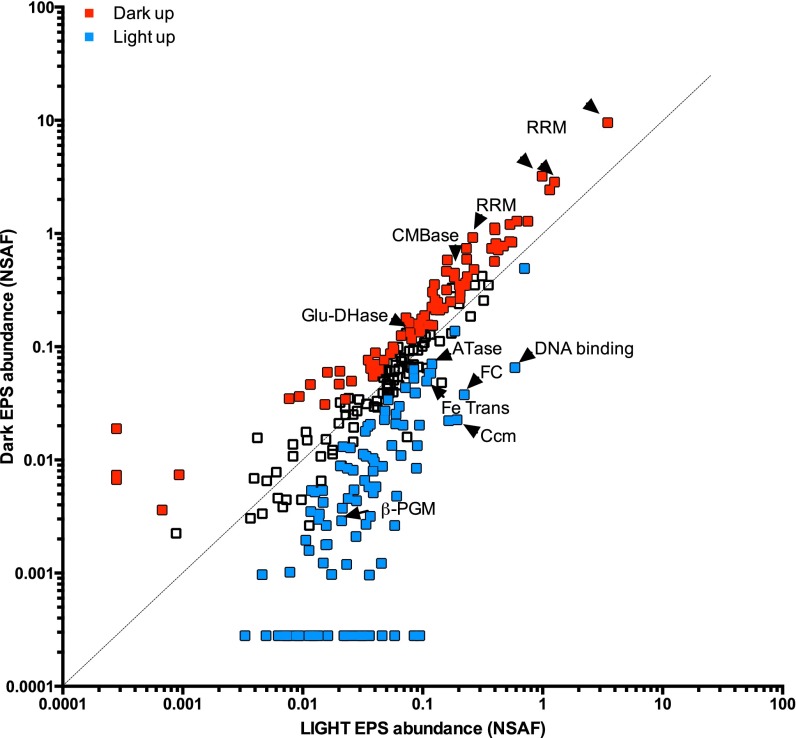
Comparison of the relative abundances determined by shotgun proteomics for the ESFC-1 exoproteome in dark versus light treatments after 3 days. Points represent exoprotein average relative abundances in comparisons between results from 3 and 5 biological replicates and 1 technical replicate (4 to 10 replicates). Relative abundance data were determined from the counts of normalized spectral abundance factors (NSAF). Exoproteins are proteins significantly enriched in the extracellular fractions relative to the total fraction (*t* test, *P* < 0.05, log_2_ fold change > 0.8). Colored points represent proteins with significant differences in abundance between the two treatments (*t* test, *P* < 0.05). CMBase, carboxymethylenebutenolidase; Glu-DHase, glucose/sorbosone dehydrogenase; RRM, 4 different RRM domain proteins; ATase, aminotransferase; β-PGM, β-phosoglucomutase; Fe Trans, Fe-regulated ABC transporter ATPase unit; DNA binding, bacterial nucleoid DNA-binding protein; FC, fasciclin domain-containing protein; Ccm, carbon dioxide-concentrating-mechanism protein.

Using shotgun metaproteomics, we examined protein composition in two operationally defined fractions of the unicyanobacterial biofilms: the extracellular “EOM” fraction, which included all soluble extracellular proteins in the biofilm, and the “total” fraction containing all intracellular and extracellular proteins. We examined five replicate biofilms after 3 days of exposure of the biofilms to three light treatments. Using the cyanobacterial genome (strain ESFC-1 [29]) as a search database, a total of 1,668 unique proteins were identified with high confidence in at least 3 of 5 replicate samples (see Table S3 in the supplemental material). Among these detected proteins, we searched for those significantly overrepresented (*P* < 0.05, >0.8 log_2_ fold) in the EOM fraction relative to the total fraction. We identified 168 proteins overrepresented in the EOM fraction (exoproteins) in the continuous light treatment, 147 in the continuous dark treatment, and 248 in the diel light treatment. Of these, 87 exoproteins were overrepresented in the dark treatment relative to the diel and/or light treatments and 107 exoproteins were overrepresented in the light treatment (Fig. 5; see also Table S3).

Exoproteins overrepresented in the continuous dark treatment had predicted functions ranging from RNA metabolism to degradation of organic compounds to stress-related activities (see Fig. S3A in the supplemental material). Two carbon breakdown-associated exoenzymes, including a putative carboxymethylenebutenolidase (CMBase), which may function in chloroaromatic degradation, and a glucose/sorbosone dehydrogenase with a carbohydrate binding domain (CBM2), were significantly overrepresented in the dark treatment (Fig. 5; see also Table S3). Three exoproteins containing the RNA recognition motif (RRM) domain were the most abundant in the dark-treated samples (Fig. 5; see also Table S3). These RRM proteins may play a role in RNA degradation, as the biofilm extracellular RNA level was significantly lower in the continuous dark treatment (Fig. 4A).

The putative CMBase had low (≥25%) sequence identity to enzymes with validated activity, so we performed sequence-to-structure modeling to link the enzyme to its potential activity. A homology-based structural model of the ESFC-1_(A2994) gene product was constructed using the crystal structure of putative dienelactone hydrolase (gene name, *ysgA*) from *Klebsiella pneumoniae* (PDB chain 3f67_A) as the structural template. It was experimentally solved at the resolution of 1.74 Å and was identified as the closest PDB template, with 36% sequence identity to A2994. Analysis of the constructed model revealed strong conformational similarities (above 85% as measured by the LGA_S score [30]) to the cluster of CMBases (EC 3.1.1.45; see Fig. S4 in the supplemental material). Additionally, the comparison of our constructed model for the ESFC-1 gene product (A2994) with the crystal structure of a dienelactone hydrolase (PDB 4u2b) from *Pseudomonas knackmussii* revealed high conservation in residues that are critical for enzyme activity (31) (see Fig. S4B in the supplemental material). Enzymes in this cluster have general carboxyl esterase activity (32), which we detected in biofilm EOM extracts, and we measured significantly higher activity in the dark treatment than in the other light regimes (Fig. 4B). Homologs of this protein exist in more than 200 cyanobacterial genomes, and many have multiple paralogs (IMG portal, http://img.jgi.doe.gov).

The continuous-light-treatment exoprotein pool included proteins with predicted functions in transport, CO_2_ concentration, and breakdown of carbohydrate, nucleic acid, and protein (see Fig. S3B in the supplemental material). This includes an Fe^3+^ ABC transporter, as well as an abundant fasciclin domain protein that may be involved in high-affinity CO_2_ uptake (Fig. 5; see also Table S3). Enzymes that may be involved in polysaccharide modification such as beta-phosphoglucomutase and 1,4-alpha-glucan branching were also overrepresented (see Table S3).

To examine the more complex “total” fraction samples, a second (liquid chromatography-tandem mass spectrometry [LC-MS/MS]) analysis was conducted (“total” analysis). For this analysis, we used iTraq isobaric tags to improve relative quantitation (33). We identified 1,838 proteins with high confidence that matched cyanobacterium ESFC-1 in at least 2 of 5 replicate samples. In the dark treatment, there was notable upregulation of a glycolysis protein, a multidrug transporter, and two stress response proteins (see Fig. S3C and S5 in the supplemental material). In the light treatment, the cyanobacterium upregulated transport of Fe^3+^, phosphate/phosphonate, and amino acids (see Fig. S3D and Fig. S5).

### Heterotroph diversity and protein expression under light, dark, and diel conditions.

**FIG 6:**
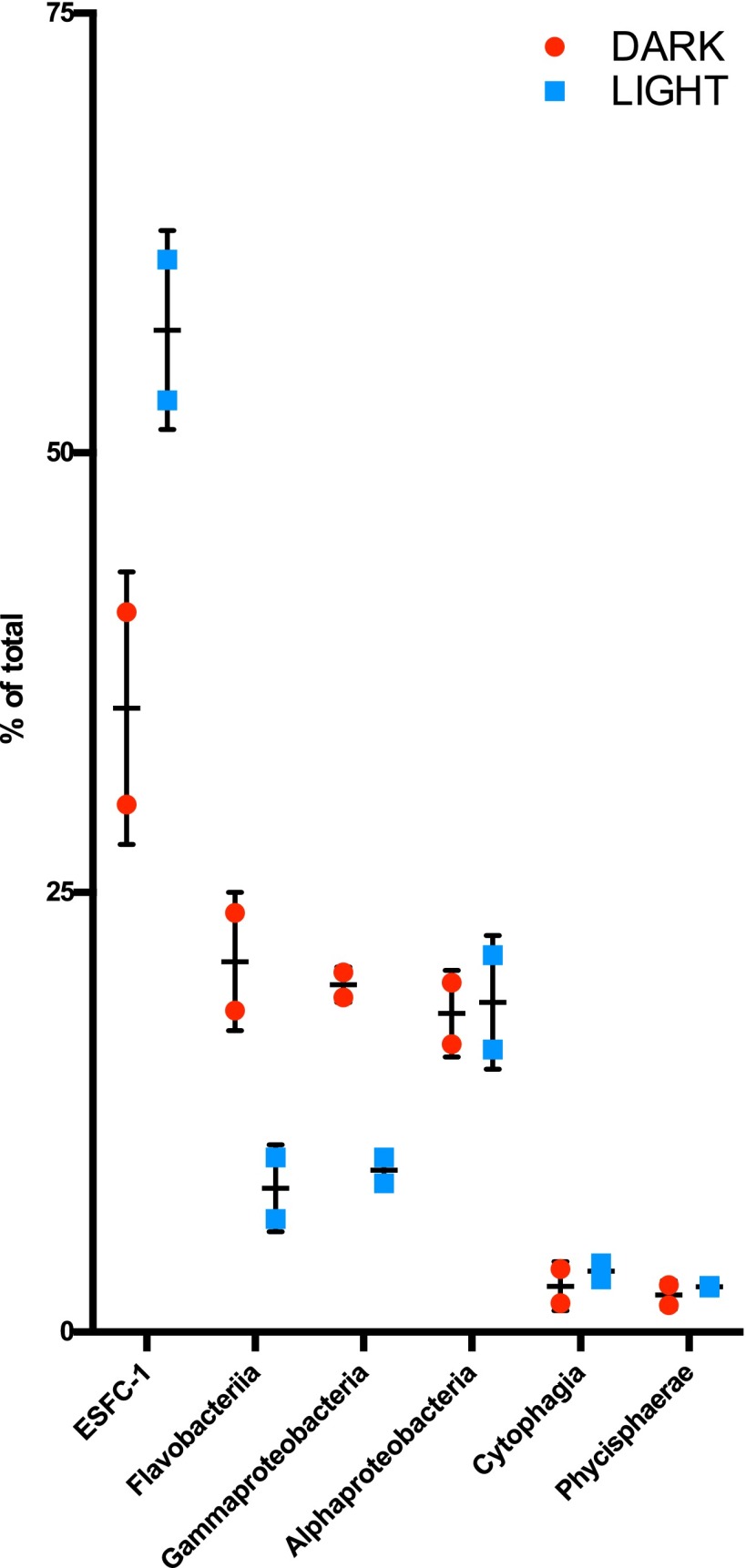
Community composition at the class level of unicyanobacterial ESFC-1 biofilms after 3 days in either continuous dark or continuous light. Points represent combined OTU percent counts of total rarefied counts: 3 OTUs for *Flavobacteriia*, 2 OTUs for gammaproteobacteria, 10 OTUs for alphaproteobacteria, and 1 OTU for each ESFC-1, *Cytophagia*, and *Phycisphaerae*. Error bars represent 1 standard deviation for comparisons between results from the two replicates.

As the identities of associated cells (referred to as heterotrophs) in this unicyanobacterial biofilm were unknown, we sequenced the 16S V4 region rRNA from the culture to identify the noncyanobacterial community and to compare levels of microbial diversity between biofilms in light and dark treatments. Four cultures were sequenced, two from a 3-day continuous light treatment and two from a 3-day continuous dark treatment. The communities consisted of 18 operational taxonomic units (OTUs) with abundance above 1% (representing the percentage of total rarefied counts), representing 4 phyla and 6 classes (see Table S4 in the supplemental material). The average relative abundance seen with members of two classes, *Flavobacteriia* and *Gammaproteobacteria*, was higher in the 3-day dark treatment than in the light treatment. We determined that the cyanobacterial OTUs in samples treated in the dark were lower in average relative abundance than those in samples treated in the light and that the members of the three other classes, *Alphaproteobacteria*, *Phycisphaerae*, and *Cytophagia*, maintained similar relative abundances between the two treatments (Fig. 6).

**FIG 7:**
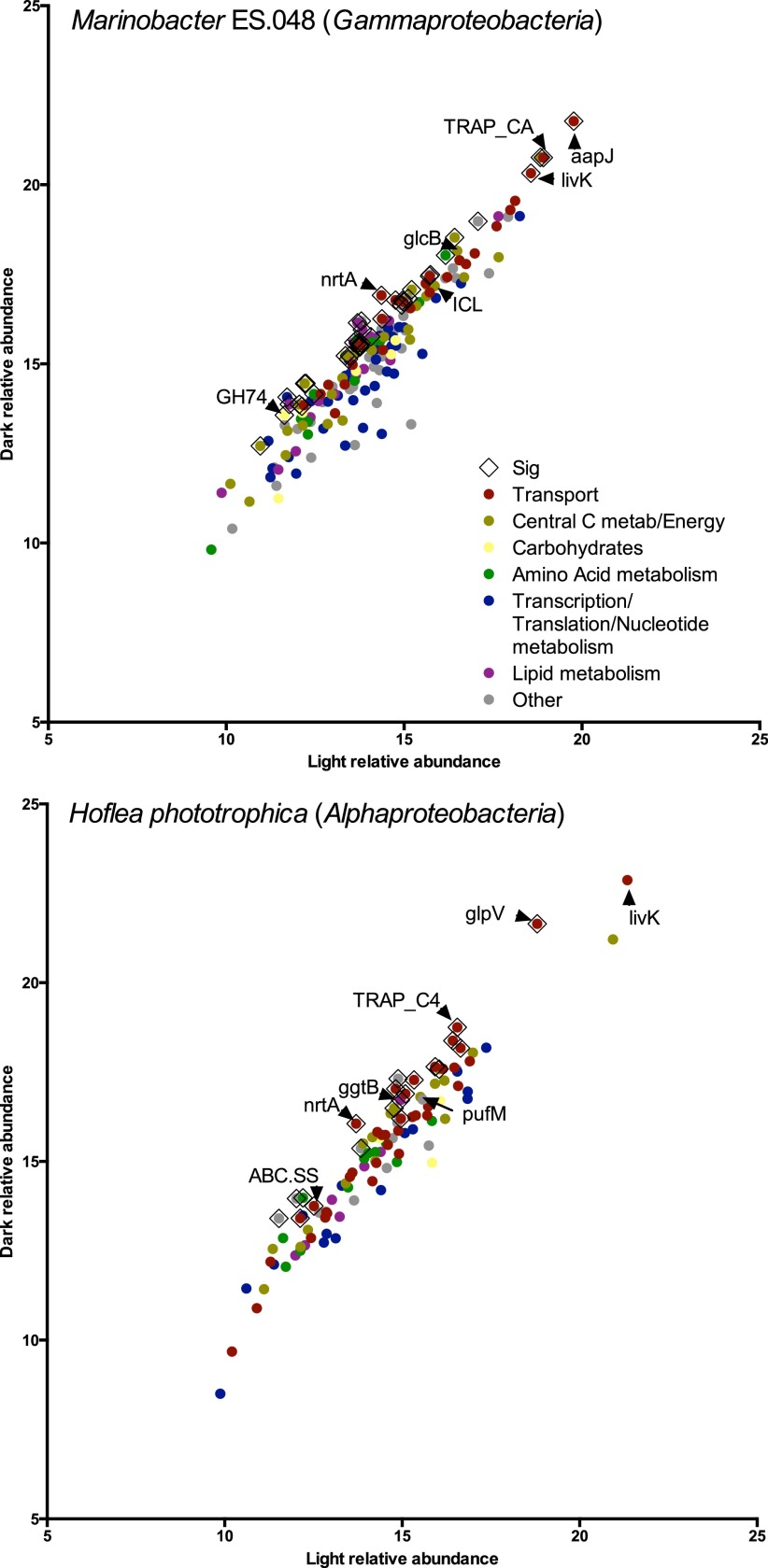
Heterotroph total (intracellular and extracellular) proteome; comparison between light and dark treatments after 3 days. Total proteome relative abundances were determined via the use of iTraq isobaric tags; points represent log_2_ normalized median-centered averages of results from five biological replicates. (Top panel) Proteins identified using *Marinobacter* sp. strain ES.048 genome. (Bottom panel) Proteins identified using *H. phototrophica* genome. Points with black diamond border indicate significant differences in average abundances (>2 standard deviations from mean difference). Colored points indicate assigned functional categories for each protein. The “Other” category includes photosynthesis, stress, motility, cell envelope, replication/recombination, and inorganic ion metabolism. aapJ, amino acid ABC transporter substrate-binding protein; livK, an ABC-type branched-chain amino acid transporter; TRAP_CA, TRAP-type mannitol/chloroaromatic compound transport system; ICL, isocitrate lyase; glcB, malate synthase; nrtA, ABC-type nitrate-nitrite transport system protein; GH74, putative endoglucanase; glpV, ABC-type glycerol transport system; TRAP_C4, TRAP-type C4-dicarboxylate transport system; pufM, photosynthetic reaction center M subunit protein; ggtB, ABC-type alpha-glucoside transport system; ABC.SS, ABC-type simple sugar transport system.

To identify proteins from the heterotroph community, we used a constructed database with 7 genomes that was based on close hits to abundant OTUs. Since our initial shotgun proteomics analysis did not yield a significant number of exoproteins from 1 of the 6 heterotroph genomes, we examined the “total” analysis. Of the 2,267 proteins identified, 429 matched one of the heterotroph genomes, while the rest were from the cyanobacterial genome (Fig. 7; see also Table S3 in the supplemental material). We refer to these 429 noncyanobacterial proteins as “heterotroph proteins.” Using a 2-sigma significance test to compare relative protein abundance levels between treatments, we identified 81 heterotroph proteins that were enriched in the dark relative to either the light treatment or the diel treatment and 5 heterotroph proteins that were overrepresented in the light treatment.

Transport and central C metabolism proteins were the most abundant heterotroph proteins detected, with many of these upregulated in the dark (see Fig. S3E and F and Table S3 in the supplemental material). We detected the highest number of heterotroph proteins (208 of the 429 heterotroph proteins; see Table S3) from one particular group (closest genome match to *Marinobacter* sp. strain ES.048). This one group represented 7% to 18% of all OTUs. Amino acid transporters were the most abundant *Marinobacter* proteins and were upregulated in the dark treatment (Fig. 7, top panel)*.* Dicarboxylate transport and metabolism proteins, including formate dehydrogenase, anaerobic selenocysteine dehydrogenase, and glyoxylate cycle proteins, were also abundant, as was a dark treatment-upregulated chloroaromatic transporter (Fig. 7, top panel; see also Table S3).

Other heterotroph proteins detected were associated with alphaproteobacterium *Hoeflea phototrophica* and with *Flavobacterium muricauda* ES.050 (*Muricauda*) (111 and 63 proteins, respectively). *H. phototrophica* expressed amino acid and dicarboxylate transporters, as well as abundant carbohydrate and sugar transporters and chloroaromatic transporters (Fig. 7, bottom panel). *H. phototrophica* also expressed an anoxygenic photosynthesis protein, PufM, at similar abundances in the light and dark treatments and upregulated an Fe^3+^ transport protein in the light treatment relative to the diel treatment (see Table S3 in the supplemental material).

## DISCUSSION

**FIG 8:**
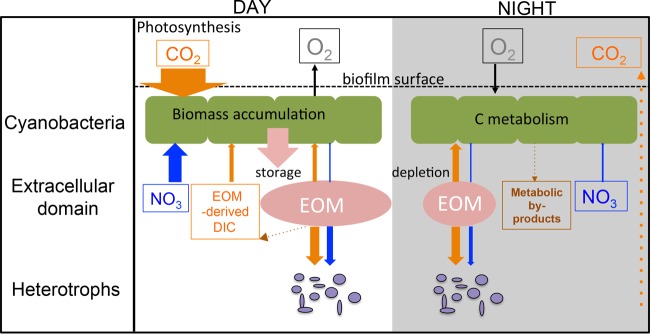
Schematic cycling of EOM in ESFC-1 biofilms following different light regimes. The widths of the arrows represent approximate levels of C or N uptake in micrograms. Orange arrows represent C flux, and blue arrows represent N flux. Dashed arrows indicate processes that were not measured or estimated.

Cyanobacteria are globally important primary producers, but the fate of their fixed carbon is complicated by their excretion, degradation, and incorporation of extracellular organic matter (EOM), the rates and pathways of which are not well constrained. In previous work, we found that a filamentous cyanobacterium (ESFC-1) in a biofilm degrades and reincorporates its own extracellular organic carbon (26). This led us to hypothesize that the cyanobacterial biofilm extracellular matrix was, among other things, a storage reservoir for C. To test this, here we compared EOM incorporation, composition, and enzymatic activity in a unicyanobacterial biofilm under continuous light (C replete; DIC through photosynthesis), continuous dark (C starved), and DCMU (oxygenic photosynthesis inhibition) treatment conditions. Our study demonstrated distinct light-dependent cyanobacterial EOM management patterns, with storage of C in the extracellular reservoir, metabolic usage at night, and micronutrient demand driving daytime incorporation (Fig. 8). Our results also suggest that community interactions play a role in EOM cycling.

### Cyanobacterial nighttime reuse of EOM C stores.

On the basis of previous work, we hypothesized that cyanobacteria would use EOM as an extracellular reserve of C for nighttime fermentation. The results of this study support this hypothesis. From flux estimates, we saw assimilation of C-rich EOM in the dark (Fig. 8), and in continuous darkness, with no other source of C, cyanobacteria showed assimilation of EOM that decreased after 6 h, which we interpret as reflecting progressive consumption of readily available EOM. This is consistent with metabolic shifts observed in natural mats, where nighttime cyanobacterial fermentation leads to sharp increases in levels of organic acids, by-products of fermentation that are thought to be excreted by cyanobacteria and utilized by other community members (34, 35). Marine picocyanobacteria in mixed assemblages also incorporate carbon from their own exudates in the dark (36), confirming that this applies to cyanobacteria in other systems. From our previous work, we know that EOM in natural mats and ESFC-1 biofilms is predominantly composed of alpha- and beta-linked glucose oligosaccharides, with smaller amounts of protein and nucleic acids (26). Our C/N relative assimilation analysis demonstrated that, in the dark, organic C was preferentially incorporated from EOM. This could indicate assimilation of oligosaccharides or assimilation of a specific compound with a higher C/N ratio than was seen with the bulk EOM.

Bulk EPS concentrations and degradation activity also supported the hypothesis that the extracellular space is used for cyanobacterial C storage. Our results indicate that EPS accumulation was light dependent in these biofilms, with high levels in the daytime and low levels at night. This is consistent with what has been observed in a benthic diatom, *Cylindrotheca closterium* (37), where EPS levels decreased in the dark. The increase of EPS levels in the light is also suggestive of a C-sink into exopolysaccharides, as occurs in species of *Nostoc* (9). Notably, extracellular proteolytic activity and carboxyl esterase activity were detectable in the light and the dark but were higher in the dark, representing a potential mechanism for usage of the extracellular stores when necessary through breakdown and uptake of degraded products.

Further evidence for this breakdown mechanism could be seen in the cyanobacterial dark-induced exoproteome. Dark-treatment-upregulated exoproteins included enzymes that may function in organic C breakdown and acquisition, which is consistent with both the C-rich incorporation data and the decreased levels of carbohydrate and exopolysaccharides in the dark treatment. Of particular interest was an abundant carboxymethylenebutenolidase (CMBase), which, according to our structural modeling, could be the main source of the observed increase in carboxyl esterase activity in the dark and may function in chloroaromatic breakdown (38). Cyanobacterial exoproteomes have been characterized for *Nostoc punctiforme* PCC 73102, *Anabaena* sp. strain PCC 7120, *Synechocystis* sp. strain PCC 6803, and eight marine *Synechococcus* strains, but CMBase was not detected in these exoproteomes (14, 39–41). However, this enzyme was detected in exoproteomes from Elkhorn Slough microbial mats (26), the ecosystem from which our cyanobacterial strain originated. Interestingly, intact mats have the ability to degrade 2,4-dichlorophenoxyacetic acid and respond to increased levels of this substrate with increased degradation (42). In our cyanobacterial biofilms, it may have a more general function in restructuring or breaking down EOM by-products.

### Daytime cyanobacterial EOM reuse for DIC and micronutrient acquisition.

In these experiments, the cyanobacteria assimilated higher and steadily increasing levels of isotope from the EOM during light periods in comparison to dark periods (Fig. 2). These results are in part explained by the fact that daytime data reflect both DIC and organic C uptake. We can disentangle these two processes with the results of our DCMU photosynthesis inhibition experiment. On the basis of those results, we estimate that at 6 h, approximately 50% of cyanobacterial ^13^C incorporation was from organic C and 50% was from DIC (Fig. 8). Using the rate of carbon fixation from our ^13^C-DIC experiments, we estimate that approximately 9% of C in the cyanobacteria was from remineralized EOM incorporation at 6 h (Fig. 8).

Past 12 h, the relative uptake of C from the EOM increased with continuous light in comparison to the N results (Fig. 2). We interpret this result as reflecting an increasing proportion of fixed DIC coming from the added EOM following prolonged light exposure, but since the continuation of DCMU experiments past 6 h is not feasible because of cell death, we cannot confirm this. Prolonged light conditions did increase the pH in the spent media (see Table S2 in the supplemental material) and led to an increase of levels of CO_2_-concentrating exoproteins, which may indicate lower availability of DIC. This suggests that cyanobacteria may preferentially acquire locally remineralized EOM in response to prolonged light exposure.

We estimate that the use of EOM in the other 50% of cyanobacteria under daytime conditions takes place by direct uptake of organic matter (Fig. 8). We hypothesize that the reason that the cyanobacteria take up this organic matter when they are fixing C is that it provides other required nutrients, such as N, P, and Fe, and that the uptake of C, for example, by organic siderophores, is incidental (43). On the basis of the photosynthesis inhibition experiment, this organic matter is N rich, contrasting with the C-rich nighttime incorporation (Fig. 8), which supports this hypothesis. We estimate that EOM was a substantial source of N for cyanobacteria in the light treatment, representing 10% of N assimilation. Uptake and assimilation of nitrate, which was provided in excess, have additional metabolic costs (44, 45), so alternative nitrogen sources (ammonium, amino acids) are often preferred. This hypothesis is further supported by evidence of increased capacity for nutrient uptake in the light. For example, ferric iron transport, phosphate/phosphonate transport, and amino acid transport were all upregulated in the light treatment, suggesting that there may have been increased demands for multiple nutrients, many of which could be counteracted through reuse of EOM. The higher abundance of exoenzymes involved in breakdown of nucleic acids and proteins, perhaps related to acquisition of phosphate (46) and N (47), supports the hypothesis that light-induced organic C incorporation is driven by a need for other micronutrients.

### Resource partitioning and interactions with heterotrophs.

Our data provide evidence for both competition and cooperation between heterotrophs and cyanobacteria. Although they constituted only 4% of the biomass, heterotrophs accounted for more than half the C and N uptake from the EOM (Fig. 8). Although we did not determine the compounds used by the two groups, it is clear from our flux estimates that they are competitors for the cyanobacteria-derived EOM (Fig. 8). In accordance with the analysis described in the previous section, the two groups were incorporating EOM with similar C/N ratios, indicating that they both target the nutrients and also express amino acid transporters at high abundances. A form of cooperation is indicated by the EOM-derived DIC used by the cyanobacteria, which likely originates from respiration by the heterotrophs, as suggested for heterotrophs associated with *Anabaena* (see, for example, reference 48). This highlights the multifactorial nature of these interactions.

We also observed changes in the heterotroph community and in the abundances of cyanobacteria relative to heterotrophs with 3 days of darkness compared to continuous light. This change in the heterotroph community was primarily due to the members of two dominant classes, *Gammaproteobacteria* and *Flavobacteria* (Fig. 6). There is evidence in diatoms of C substrate exchange for Fe from associated *Marinobacter* species (49), as well as in targeted feeding of sulfonate compounds to a marine *Roseobacter* in exchange for vitamin B_12_ (50), both secretion mechanisms by which the primary producers can control heterotroph populations. Due to their close spatial relationships with other community members, mat cyanobacteria may be particularly suited to controlling the availability of extracellular compounds to heterotrophs, through uptake as well as secretion. Recent work has shown excretion of a much wider range of metabolites by a mat cyanobacterium, *Microcoleus vaginatus*, than by an aquatic cyanobacterium, *Synechocystis* sp. strain PCC 6803, and reuptake by *Microcoleus* of a large number of those metabolites (51), suggesting that mat organisms may tailor their immediate extracellular environment. In addition to the hypothesis that cyanobacteria outcompete the heterotrophs for certain compounds, they could also be excreting allelopathic compounds, an activity which would be discontinued in the dark due to C limitation.

### Heterotroph metaproteome expression.

By comparing proteomes from several groups of heterotrophs, we found that the heterotrophs may have distinct growth strategies for using cyanobacterial products that allow them to circumvent heterotroph-heterotroph competition and to respond to the changing light regimes. The proteomic response from our representative gammaproteobacterium (*Marinobacter* sp. strain ES.048) suggested a specialist strategy, utilizing amino acids and simple carbon compounds through the glyoxylate pathway. Experimental evidence and genomic predictions support these specializations for this group (47, 52). *Marinobacter* also upregulated sugar alcohol and chloroaromatic transport proteins in the dark, suggesting that this group may take advantage of cyanobacterial dark extracellular degradation, given the upregulation of extracellular cyanobacterial CMBase in the dark treatment. In contrast, the proteomic response from our representative *Rhizobiales* genome, *H. phototrophica*, indicates a more generalist strategy that includes incorporation of a wide range of compounds, supplemented by anoxygenic photosynthesis. For example, *H. phototrophica* expressed transporter proteins responsible for uptake of carbohydrates, sugars, osmolytes, organic acids, and citrate, which were not detected in *Marinobacter*, as well as nitrate, amino acid, dicarboxylate transport, and chloroaromatics, which were found in both classes. The *H. phototrophica* genome also has a full set of genes for anoxygenic photosynthesis (53), and at least one of these photosynthesis proteins was expressed. This may explain why this group did not increase its relative abundance in the dark treatment, since some members may rely on energy from light. Work on other unicyanobacterial consortia with similar levels of heterotroph taxonomic diversity shows successional species composition over biofilm development (54), and this may be related to the availability of extracellular resources. Our work builds upon those findings by describing proteomic responses of some of these groups to changing resources, which we controlled by extended incubations in treatments using continuous light and continuous darkness. The differences in responses between groups provide insight into the partitioning of extracellular resources in this community.

### Conclusions.

In summary, cyanobacteria not only produce EOM but also use it directly both in the light and in the dark (Fig. 8). It is likely that cyanobacteria also benefit from the remineralization of EOM by heterotrophic bacteria during periods of photosynthesis. Our biofilm cyanobacterium accounted for 43% of the total EOM C assimilation in the dark and appeared to metabolize extracellular resources under C-starved conditions. On the basis of the proteome and the C/N content of the EOM incorporated by cyanobacteria in the light treatment, it seems likely that light-driven incorporation occurs primarily in acquisition of micronutrients. Furthermore, cyanobacteria produce degradation exoenzymes and the heterotrophs respond with upregulation of transporters, suggesting the presence of a dynamic extracellular pool requiring specialized transport. While primary producer excretions are acknowledged to influence microbial organic matter cycling in many systems, the redirection of complex organic carbon back to the primary producer is not nearly as well documented. This redirection has the potential to substantially influence EOM composition and remineralization rates, and our data suggest that determining primary producer metabolic status is critical for a complete understanding of organic matter turnover in these systems.

## MATERIALS AND METHODS

### Cell culture and extracellular separation.

Unicyanobacterial cultures of the cyanobacterium ESFC-1 were grown as described by Stuart et al. (26) in artificial seawater media (ASN) (27, 55) at 23°C with 20 µmol m^−2^ s^−1^ (4.16 W m^−2^) light on a 12-h/12-h light/dark cycle. Extraction of EOM from the biofilms was carried out as described by Stuart et al. (26). Briefly, biofilms were weighed and homogenized on ice in sterile 10% NaCl (Wheaton Dounce homogenizer; pestle clearance, 0.114 ± 0.025 mm). Homogenate was incubated at 40°C for 15 min and centrifuged at 15,000 × *g* at 4°C for 20 min and supernatant filtered through a 0.2-µm-pore-size polycarbonate filter, to separate out cells, forming the extracellular (or “EOM”) fraction.

### ^13^C-labeled and ^15^N-labeled extracellular organic fraction.

^13^C-labeled and ^15^N-labeled EOM was generated as described by Stuart et al. (26), excepting that 1.76 mM K^15^NO_3_ (Sigma-Aldrich Co., St. Louis, MO, USA) (98 atom percent ^15^N) was substituted in ASN, along with 2 mM ^13^C sodium bicarbonate (Cambridge Isotopes, Tewksbury, MA, USA) (^13^C; 99%). Bulk isotope ratios for ^13^C/^12^C and ^15^N/^14^N of a sample of ^13^C-labeled and ^15^N-labeled substrate were determined by automated nitrogen and carbon analysis-IRMS (ANCA-IRMS) (PDZE Europa Limited, Crewe, England) at the University of California, Berkeley.

### Reuptake of EOM.

To quantify differences in uptake of extracellular material in the cyanobacterium and heterotrophs under various light conditions, replicate unicyanobacterial biofilms were grown as a biofilm for 1 month in 13 6-well plates as described by Stuart et al. (26). After 20 days of growth, a total of six treatments were applied: “killed,” “no label,” “dark,” “diel,” “light,” and “DCMU.” “Killed” controls were generated by addition of 4% formaldehyde to wells and incubation overnight before the experiment start. At the start of the light phase of the 12:12 diel incubation cycle, medium was then removed from all cultures and 3 ml of ^13^C-labeled and ^15^N-labeled EOM (described above) diluted 1:30 in fresh sterile medium was added to each well. For “no-label” control, only fresh sterile medium was added. For “dark” treatments, 4 plates were wrapped in 2 layers of tin foil and incubated with the 4 “diel” plates under conditions of 12-h/12-h light/dark. The cultures receiving the “light” treatment were moved to a constant-light incubator from the 12-h time period to the 24-h time period following the addition. For the “DCMU” treatment, 3-(3,4-dichlorophenyl)-1,1-dimethylurea (DCMU) was added at 20 µM along with the labeled substrate addition and the plate was incubated in the light. At 2, 6, and 12 and 24 h, three replicate cultures from each of the dark and diel treatments were washed with sterile unlabeled ASN, incubated with 4% formaldehyde for 1 to 2 h, rinsed with 1× phosphate-buffered saline (PBS), and frozen at −20°C in 50% ethanol. At the 6-h time point, killed, no-label, and DCMU control cultures were also fixed as described above. The cultures that received light treatment were also fixed at the 24-h time point as described above. To prepare these samples for NanoSIMS analysis, subsamples were removed, homogenized gently to separate trichomes, and rinsed with sterile MilliQ water three times to remove unincorporated label. A 0.5-µl volume was then spotted onto a silica wafer, air dried, and stored in an argon dry box.

Parallel ^13^C bicarbonate additions (2 mM) were run for 6 h on 9 replicate biofilms, using 3 replicates each for a dark incubation, a lighted incubation, and a killed control. Biofilms were fixed as described above and bulk isotope ratios determined by IRMS as described above.

### NanoSIMS isotope imaging and scanning electron microscopy (SEM).

Samples were prepared as described by Stuart et al. (26). Briefly, wafers were coated with ~5 nm of gold, and secondary ion mass spectrometry (SIMS) imaging was performed with a Cameca high-resolution imaging SIMS (NanoSIMS) 50 microprobe at Lawrence Livermore National Laboratory. A focused primary ion beam (2 pA, approximately 150 nm, 16 keV ^133^Cs^+^) was used in a raster pattern with 15-by-15 µm^2^ to 25-by-25 µm^2^ analysis areas of 256-by-256 pixels and a dwell time of 1 ms/pixel for 19 to 30 scans (cycles). Before analysis, samples were presputtered with 90 pA of Cs^+^ current (equivalent to approximately 50 nm) to reach sputtering equilibrium and to make sure that the isotope analysis targeted intracellular material rather than the surface of the cells. Serial quantitative secondary ion images (maps) were simultaneously collected for ^12^C_2_^−^, ^13^C^12^C^−^, ^12^C^14^N^−^, and ^12^C^15^N^−^ using electron multipliers in pulse counting mode. Previous work indicated that the C dimer (e.g., ^12^C^12^C) provides yield superior to that of the ^12^C monomer (56). Secondary electrons were also simultaneously collected as previously described (56).

NanoSIMS ion image data were processed as described by Woebken et al. (27). For each raster, quantitative ion ratio images were generated from the summed ion images to generate ^13^C and ^15^N enrichment images, presented in values corresponding to atom percent excess (APE) (56, 57). Regions of interest (ROIs) for quantification of isotopic ratios were selected on the basis of secondary electron images, ^13^C enrichment images, and ^12^C^14^N^−^ ion images, which allowed cells to be specifically selected and hot spots of residual labeled substrate to be excluded. Isotopic ratios were extracted per cycle and averaged.

### Biovolume, net fixation, and C/N substrate usage calculations.

Bacterial counts and biovolume measurements were done for all biofilm samples analyzed with NanoSIMS, as described by Stuart et al. (26). Briefly, biofilms were imaged, images were analyzed to calculate biovolume, and C content was estimated using biovolume-to-carbon conversion factors from the literature (2.2 × 10^−13^ g C µm^−3^ for other bacteria and 1.8 × 10^−13^ g C µm^−3^ for filamentous diazotrophic cyanobacteria) (58, 59). Cyanobacterial biovolumes within the biofilms were not significantly different between treatments (5.57 × 10^8^ µm^−3^/well ± 1.55 × 10^8^ µm^−3^/well, 5.61 × 10^8^ µm^−3^/well ± 1.55 × 10^8^ µm^−3^/well, and 5.95 × 10^8^ µm^−3^/well ± 3.29 × 10^8^ µm^−3^/well for 24-h dark treatment, light treatment, and killed control, respectively). N content was estimated based on C/N ratios determined via elemental analysis of biomass coupled with IRMS analysis and determined from the literature for marine bacteria (C/N of 4.0 for cyanobacteria and 4.4 for heterotrophs) (60). C and N content was extrapolated to micrograms of C or N per well for the 6-well plates used. ^13^C and ^15^N APE values were compared between treatments and time points by use of one-way analysis of variance (ANOVA) and Dunnett’s *post hoc* test for comparison with killed controls and Tukey’s *post hoc* test for comparison between time points within a given treatment. Net fixation of ^13^C and ^15^N (percentage of C or N incorporated relative to initial C or N content) (28) was calculated for both heterotrophs and cyanobacteria and extrapolated to calculate micrograms of C or N fixation per well. C/N relative use efficiency data were calculated as described by Mayali et al. (28), and mean use efficiencies were compared to a theoretical mean of 1.0 using a *t* test.

### Bulk composition and enzyme activity assays and oxygen measurements.

Batch cultures were inoculated into 300 ml of ASN in acid-washed 1-liter glass flasks and grown as described by Stuart et al. (26). At 4 weeks, at the onset of the light phase of the diel cycle, 3 treatments (dark, light, and diel treatments) were initiated in 3 replicate flasks (9 total). Dark-treated biofilms were incubated in the dark for 3 days, light-treated biofilms were incubated under constant illumination for 3 days, and diel-treated biofilms were incubated using a 12-h/12-h light/dark cycle. After 3 days of treatment, all biofilms were harvested and the extracellular fraction was separated as described above. Carbohydrate concentration was determined using the traditional phenol sulfuric acid method (61). Nucleic acid concentrations were measured using PicoGreen and RiboGreen dyes for DNA and RNA, respectively, according to instructions from the manufacturer (Life Technologies, Carlsbad, CA). Protein concentrations were determined using a Bradford assay (Bio-Rad, Hercules, CA) and absorbance at 280 nm. All concentrations were normalized to biomass wet weight.

Enzyme activities were assayed in the extracellular fraction following the protocol established by Bell et al. (62) and described by Stuart et al. (26)*.* Briefly, diluted extracellular fraction samples were combined with a fluorescent substrate. l-Leucine-7-amido-4-methylcoumarin hydrochloride, 4-methylumbelliferyl α-d-glucopyranoside, 4-methylumbelliferyl β-d-glucopyranoside (Sigma Aldrich, St. Louis, MO, USA), and 4-methylumbelliferyl caprylate (Santa Cruz Biotechnology, Santa Cruz, CA, USA) were the substrates for peptidase, α-glucosidase, β-glucosidase, and carboxyl esterase activity, respectively. Controls included each sample with water and sterile 10% NaCl (substrate control) instead of sample. Plates were read every 5 min for 3 h at 380/440 nm.

Congo red staining was performed on biofilms grown for 5 days on glass chamber slides with 3 ml of ASN. Triplicate biofilms were then incubated under dark, diel, or full-light conditions for 3 days and fixed with 4% formaldehyde at 4°C. Biofilms were rinsed with sterile water, stained with DAPI (4′,6-diamidino-2-phenylindole) for 10 min at 23°C (10 µg/ml), rinsed again, and then stained with Congo red (1 mg/ml) for 15 min at 23°C. Biofilms were then rinsed with 1 M NaCl (added to remove excess stain). A total of 10 fields of view for each biofilm were imaged in 9-µm z-stacks in both the Cy3 and DAPI channels at 100% power using a 150-ms exposure. Images were then combined into a maximum projection image, and cyanobacterial filaments were manually outlined. Fluorescence intensity was then quantified, excluding the filaments. Mean intensities were compared using one-way ANOVA and Tukey *post hoc* tests.

Oxygen measurements were performed on biofilms grown in 6-well plates as described above with triplicate wells for three treatments: light, dark, and diel. After 3 days of treatment, oxygen was measured at the surface of each biofilm using an OXR50 optical oxygen sensor coupled to a PyroScience FireSting optical oxygen meter (Pyro Science GmbH, Aachen, Germany).

### 16S iTag sequencing.

Illumina MiSeq sequencing was done at Laragen (Culver City, CA) using primers for amplification of the 16S V4 region (obtained from Caporaso et al. [63]). Analysis details are described in Text S1 in the supplemental material.

### LC-MS/MS metaproteomics.

Batch cultures were grown and the extracellular “EOM” fraction was extracted as described above, with 5 replicates per treatment (total, 15 flasks). Additionally, following homogenization, a 1/10 vol of the homogenate from each biofilm was separated, sonicated to lyse cells (Misonix, Farmingdale, NY; 50% intensity, 6 cycles of 30 s on and 2 min off on ice), and centrifuged at 12,000 × *g* at 4°C for 10 min. The supernatant constituted the “total” fraction and included both intracellular and extracellular proteins. This resulted in 30 total samples for proteomic analyses. Run details are described in Text S1 in the supplemental material.

### Metaproteomic analyses.

The MSGF+ search algorithm was used to match the spectra to peptide sequences (64) derived from the ESFC-1 genome (61) or a constructed database as described below, each amended with common contaminant sequences (keratins, trypsin, serum albumins, etc.); the search parameters included partially tryptic cleavages, dynamic modification of methionine oxidation, and a ± 20 ppm parent ion mass tolerance.

To identify proteins expressed by the heterotrophic population, we identified genomes with high BLASTn identity scores to abundant OTUs from our iTag data and constructed a search database to query the spectra of the iTraq isobaric tags. The gammaproteobacterial OTU with the highest abundance matched an Elkhorn Slough microbial mat isolate, *Marinobacter* sp. strain ES.048. For the 10 OTUs in the alphaproteobacterial class, we chose two genomes that had the highest identity (100% and 99% at the 16S rRNA gene level) to 2 alphaproteobacterial OTUs, from the *Rhizobiales* (*H. phototrophica* DFL-43) and *Rhodobacter* (*Tropicibacter multivorans* DSM 26470) orders, respectively. The *Flavobacteriia* class was represented by 3 OTUs, and the OTU with the highest abundance did not match any database genome 16S sequence above 90% identity, so we used the next-most-abundant OTU, which matched a bacterium isolated from Elkhorn Slough, *Muricauda* sp. strain ES.050, with 100% identity. The *Phycisphaerae* and *Cytophagia* classes were represented by 1 OTU each, so we selected the most closely related genomes, *Tepidiphilus thermophilus* JCM 19170 and *Algoriphagus terrigena* DSM 22685, which had 81% identity and 94% identity to their respective OTUs. We constructed a database using these 6 genomes and the ESFC-1 genome and used it for comparisons to our proteomics samples.

Reporter ion abundance values were extracted using MASIC (65), and their respective sequences were aggregated using an in-house processing pipeline, including the APE software package (http://omics.pnl.gov/software/ape-mdart) with a 1% false discovery rate (FDR) cutoff (MSGF + Q-value ≤ 0.01). Peptide data were combined to protein levels using summed abundances, which were subsequently log_2_ normalized and zero centered. The significance of data corresponding to protein abundances across treatments was determined based on a 2 sigma deviation (*P* value, ≤0.05). Cutoffs of at least 2 unique peptides and presence in at least three biological replicates in a given treatment were applied.

Spectral counts were used to generate normalized spectral abundance factors (NSAF) (66), generating relative percent abundances, normalized to protein length for each protein identified. For proteins that were not detected in some of the replicates, we substituted a value of one-quarter of the lowest NSAF calculated (67). We then generated average abundances for proteins found in at least 3 biological replicates and at least 4 of the 10 total replicates (5 biological and 2 technical for each treatment). We compared abundances of proteins between treatments using a Student’s *t* test. Localization predictions were made using MetaLocGramN (68) and SecretomeP (69) for nonclassical secretion prediction. CAZy enzymes were predicted using dbCAN (70).

## SUPPLEMENTAL MATERIAL

Text S1  Supplemental materials and methods. Download Text S1, DOCX file, 0.1 MB

Figure S1  ^13^C and ^15^N enrichment of cells analyzed via NanoSIMS. Each point represents ^13^C and ^15^N atom percent excess (APE) for a single trichome (solid) or bacterial cell (outlined). The dotted line indicates the ratio of the levels of ^13^C-labeled and ^15^N-labeled EOM-substrate, as determined by IRMS. Download Figure S1, EPS file, 0.2 MB

Figure S2  Exopolysaccharide stain Congo red in biofilms grown under different light regimes for 3 days. (A and B) Representative epifluorescent microscopy images of Congo red-stained continuous-light-treated biofilm (A) and continuous-dark-treated biofilm (B). The white scale bar represents 10 µm. The blue coloring is from DAPI DNA staining, and the red coloring is from both autofluorescence (inside filaments) and Congo red stain (extracellular). Yellow outlines indicate lines manually drawn to exclude cyanobacterial cells from analysis of Congo red intensities. (C) Average extracellular Congo red intensities under different light regimes for 3 days. Error bars represent 1 standard deviation of results of comparisons between 3 biological replicates (10 fields of view per replicate analyzed). *, significant difference in means (*P* < 0.05). Download Figure S2, EPS file, 13.9 MB

Figure S3  Functional predictions for proteins significantly overrepresented in either continuous dark (panels A and C and panel E) or continuous light (panels B and D and panel F) treatments. (A and B) The top panels show functional categories for ESFC-1 exoproteins overrepresented in either continuous dark (A) or continuous light (B) treatments. (C and D) Middle panels show functional categories for ESFC-1 total proteome proteins overrepresented in either continuous dark (C) or continuous light (D) treatments. (E and F) The bottom panels show functional categories for total proteome heterotroph proteins overrepresented in the continuous dark treatment from representative gammaproteobacterium *Marinobacter* sp. strain ES.048 (E) or representative alphaproteobacterium *H. phototrophica* (F). Significantly overrepresented, the abundance of the proteins was higher that seen with either the diel treatment or the light or dark treatment (*P* < 0.05). Download Figure S3, TIF file, 21.5 MB

Figure S4  Sequence-structure-based analyses of the ESFC-1 CMBase. (A) StralCP dendrogram and clusters for proteins identified in PDB with predicted structural similarity to A2994. Proteins with known enzyme activity have the EC number listed together with corresponding PDB chain identifier (ID) labels. (B) List of residue-residue correspondences between the structural model of A2994 and ClcD from *Pseudomonas knackmussii* (PDB chain 4u2b_A; resolution, 1.70 Å; sequence identity, 26%) in critical functional positions. Catalytic triad residues are colored in red. Mutation positions that improve activity toward substrates are in green. In blue are colored positions where mutations increase substrate accessibility during catalysis by increasing flexibility of the loop containing catalytic triad histidine. The distances between corresponding *C*α atoms from the calculated structure superposition are displayed in the last column. Positions marked by an asterisk (*) denote perfect agreement between residues from A2994 and native or mutated residues from 4u2bA. (C) Cartoon representation of the structural superposition of A2994 model (gray) with the crystal structure of ClcD dienelactone hydrolase (yellow). Side-chain conformations of corresponding residues identified as functionally critical are presented in “ball and stick” form using the same coloring scheme as in the table in panel B and numbering from A2994. Download Figure S4, EPS file, 2.1 MB

Figure S5  *Cyanobacteria* total (intracellular and extracellular) proteome differences between light and dark treatments after 3 days. Total proteome relative abundances were determined via the use of iTraq isobaric tags, and points represent log_2_ normalized median-centered averages of results from five biological replicates. Colored points represent proteins with significant differences in average abundance (>2 standard deviations from mean difference). pfkA, 6-phosphofructokinase; ABC.2A, ABC-type multidrug transport system; glnB, nitrogen regulatory protein P-II; AfuA, ABC-type Fe3^+^ transport system; CphX, CO2 hydration protein; phnD, ABC-type phosphate/phosphonate transport system; petJ, cytochrome c6; ABC.PA, ABC-type polar amino acid transport system. Download Figure S5, EPS file, 0.3 MB

Table S1  Number of cells analyzed via NanoSIMS. Table S1, DOCX file, 0.05 MB

Table S2  O_2_ and pH measurements. Table S2, DOCX file, 0.04 MB

Table S3  Proteomics results. Data represent exoproteins and total proteins identified in cyanobacteria and heterotrophs under all experimental conditions. Table S3, XLSX file, 1 MB

Table S4  Relative abundances of bacterial taxa based on 16S iTag sequencing in biofilms treated in the light and the dark. Table S4, DOCX file, 0.1 MB
