# Supplementary material for: Light Regimes Shape Utilization of Extracellular Organic C and N in a Cyanobacterial Biofilm
Source: mBio. 2016 Jun 28;7(3):e00650-16. doi: 10.1128/mBio.00650-16 (PMC4937211; doi:10.1128/mBio.00650-16)
Supplement: Text S1 — Supplemental materials and methods. Download [file mbo003162866s1.docx]

**Supplemental Materials**

**Supplemental Methods**

*LC-MS/MS Metaproteomics*. Urea and dithiothreitol (DTT) (all chemicals purchased from Sigma-Aldrich, St. Louis, MO, USA, unless otherwise stated) were added to the samples to bring them to a concentration of 8 M and 10 mM respectively. Samples were then mixed by sonication and vortexing, followed by a brief centrifugation to collect all liquid in the bottom of the tube. Samples were incubated at 60°C for 30 min with constant shaking at 800 rpm, followed by dilution with 1600 µL of 100 mM ammonium bicarbonate, pH 8. Calcium chloride (1 mM) was then added to the samples. Sample digestion was performed by the addition of 10 µL trypsin (Promega) (trypsin/protein, 1:50, wt/wt) to samples, which were then incubated at 37°C for 3 hrs, cleaned using a Supelco Discovery C18 solid phase extraction (SPE) column with a Gilson GX-274 ASPEC™ system (Gilson Inc., Middleton, WI, USA), eluted into a low-binding 1.5 mL centrifuge tube, and then dried to 100 µL. A bicinchoninic acid (BCA) assay was used to determine sample protein concentration (Thermo Scientific, Rockford, IL, USA). An equal concentration of protein (25 μg) was aliquoted into separate tubes after which samples were brought to a volume of 30 μl by adding 0.5 M triethylammonium bicarbonate (TEAB), or concentrated. Frozen 4-plex iTRAQ reagent vials (AB Sciex, Framingham, MA, USA) were brought to room temperature then pulse spun to collect contents at the bottom. Isopropanol in the amount of 60 μL was added to each reagent vial after which the vials were thoroughly vortexed, spun down, and added to samples. Vials were rinsed with an additional 10 μL of isopropanol which was also added to the samples. Four channels of iTRAQ reagents, ranging from 114 to 117, were used to label 5 sets of the total fraction samples. Each of the 5 replicates of the light samples were labeled with 114; diel conditions, 115; dark, 116; and a universally pooled control, 117. Sample pH was adjusted to 7.8 for the labeling reactions, with an organic concentration of at least 60% (v/v). Samples with added iTRAQ reagents were vortexed, centrifuged briefly, then incubated at room temperature for 2 hours. To stop the labeling reaction, 100 μL of nanopure water was added to each vial to hydrolyze any remaining reagent and then samples were incubated for an additional 30 min. Samples were then placed in a speed-vac to remove the organic solvent, and then pooled together within their respective sets and dried down to a final volume of ~100 μL. An SPE C18 was used to clean the final 5 samples and BCA assayed for high pH C18 HPLC fractionation.

Samples were diluted to a volume of 400 uL with 10 mm ammonium formate buffer (pH 10.0), and resolved on a XBridge C18, 250x4.6 mm, 5 μM with 4.6x20 mm guard column (Waters, Milford, MA). Separations were performed at 0.5 mL/min using an Agilent 1100 series HPLC system (Agilent Technologies, Santa Clara, CA) with mobile phases **(A)** 10 mM Ammonium Formate, pH 10.0 and **(B)** 10 mM Ammonium Formate, pH 10.0/acetonitrile (10:90). The gradient was adjusted from 100% A to 95% A over the first 10 min, 95% A to 65% A over minutes 10 to 70, 65% A to 30% A over minutes 70 to 85, maintained at 30% A over minutes 85 to 95, re-equilibrated with 100% A over minutes 95 to 105, and held at 100% A until minute 120. Fractions were collected every 1.25 minutes, (96 fractions over the entire gradient with the exception of the first 15 mins to avoid collection of residual iTRAQ label) and every 24th fraction was combined for a total of 24 samples (each with n=4 fractions pooled) for each of the 5 LC runs. All fractions were dried under vacuum and 20 uL of 25 nanopure water was added to each fraction for storage at -20°C until LC-MS/MS analysis.

A nanoAquity UPLC® system (Waters Corporation, Milford, MA) used for chromatographic separation of digested sample fractions. The HPLC column, packed in house, consisted of a 70-cm length of 360 µm o.d. x 75 µm i.d. fused silica capillary tubing (Polymicro Technologies Inc., Phoenix, AZ) packed with 3-µm Jupiter C18 stationary phase (Phenomenex, Torrence, CA). The column was equilibrated at 99 % mobile phase A (0.1% formic acid in water) and 1% mobile phase B (0.1% formic acid in acetonitrile), with a flow of 300 nL/min and the following gradient profile (min, %B): 0, 1; 2, 8; 20, 12; 75, 30; 97, 45; 100, 95; 110, 95; 115, 1; 150, 1.

The LC was coupled to a LTQ Orbitrap Velos mass spectrometer (Thermo Scientific, San Jose, CA) with custom electrospray ionization (ESI) interface, an ion transfer tube temperature of 350ºC, and spray voltage of 2.2 kV. Orbitrap spectra (AGC 1x106) were collected from 400-2000 m/z at a resolution of 60K followed by data dependent ion trap MS/MS spectra (AGC 1x104) of the ten most abundant ions using a 2 amu isolation width, 35% collision energy and activation time of 10 ms. A dynamic exclusion time of 60 sec was used to discriminate against previously analyzed ions (within -0.55 and 1.55 amu).

*Sequence-structure modeling:* A homology-based structural model of the ESFC-1 gene of interest, locus number A3MYDRAFT_2994 (A2994), was constructed using the AS2TS system (1), and the crystal structure of putative dienelactone hydrolase (gene name: ysgA) from *Klebsiella pneumoniae* was identified as the closest PDB structural template (PDB chain: 3f67_A) for the modeling. It was experimentally solved at the resolution of 1.74 Ångstroms, and the level of sequence identity between A2994 and ysgA is 36%. Another example of close structural homologs that was identified and leveraged during the modeling process include a crystal structure of an uncharacterized protein (gene: ecs3884) from *Escherichia coli* (PDB chain: 4zv9_A; resolution: 2.00 Ångstroms; sequence identity: 28%). The constructed models enabled combined sequence- and structure-based PDB searches that lead to identification of other well characterized proteins whose potential functional similarity to A2994 could not be assessed with confidence using only sequence-based approaches. To determine structural groupings and gain better insight into both overall and region-specific similarities between proteins, clustering of the structures was performed using StralCP algorithm (2).

*16S iTag sequencing.* All sequences were analyzed using USEARCH (usearch v8.0.1623), a sequence analysis tool provided by Robert Edgar (<http://drive5.com/usearch>). Barcodes and primers were already removed by the sequencing provider, Laragen. DNA reads were merged before filtering. After merging, reads were discarded that exceeded a set maximum expected errors threshold of 1 error per 100 basepairs. The resulting reads of 256 bp were used in further analysis. Singleton reads were removed. *De novo* OTU representative sequences made from USEARCH’s OTU clustering algorithm had a maximum sequence identity difference of 3% (corresponding to OTU identity threshold of 97%). Chimeric reads were automatically ignored during the OTU generation process. However, to check for chimeric OTUs that were missed, additional referenced based chimera filtering was done using UCHIME and the “Gold” database (provided at <http://drive5.com/uchime/uchime_downlpad.html>; made from the ChimeraSlayer reference database). The phylogenetic identity of the OTUs was determined using UClust in QIIME and the Greengenes taxonomy database (QIIME v1.7.0). All of the samples were rarified down to 182,085 reads.

**Supplemental References**

1. **Zemla A, Zhou CE, Slezak T, Kuczmarski T, Rama D, Torres C, Sawicka D, Barsky D.** 2005. AS2TS system for protein structure modeling and analysis. Nucleic Acids Res **33:**W111-W115.

2. **Zemla A, Geisbrecht B, Smith J, Lam M, Kirkpatrick B, Wagner M, Slezak T, Zhou CE.** 2007. STRALCP—structure alignment-based clustering of proteins. Nucleic Acids Res **35:**e150.

**Supplemental Figure and Table Legends
Figure S1**: ^13^C and ^15^N enrichment of cells analyzed via NanoSIMS. Each point represents ^13^C and ^15^N atom percent excess (APE) for a single trichome (solid) or bacterial cell (outlined). Dotted line indicates ratio of ^13^C-^15^N labeled EOM-substrate, as determined by IRMS.

**Figure S2:** Exopolysaccharide stain Congo Red in biofilms grown under different light regimes for 3 days. Representative epiflourescent microscopy images of Congo Red stained **(A)** continuous-light treated biofilm and **(B)** continuous-dark treated biofilm. White scale bar represents 10 µm. Blue color is from DAPI DNA stain and red is from both autofluorescence (inside filaments) and Congo Red stain (extracellular). Yellow outlines indicate manually drawn lines to exclude cyanobacterial cells from analysis of Congo Red intensities. **(C)** Average extracellular Congo Red intensity under different light regimes for 3 days. Error bars represent one standard deviation between 3 biological replicates (10 fields of view per replicate analyzed). “*” indicates significant difference in means (p<0.05).

**Figure S3:** Functional predictions for proteins significantly overrepresented in either continuous dark (**A, C, E**) or continuous light (**B, D, F**) treatments. Top panels show functional categories for ESFC-1 exoproteins overrepresented under either continuous dark (**A)** or continuous light **(B)**. Middle panels show functional categories for ESFC-1 total proteome proteins overrepresented under either continuous dark **(C)** or continuous light **(D).** Bottom panels show functional categories for total proteome heterotroph proteins overrepresented under the continuous dark treatment from representative Gammaproteobacterium *Marinobacter* strain ES.048 **(E)** or representative Alphaproteobacterium *H.phototrophica* (**F).** ‘Significantly overrepresented’ proteins had higher abundance relative to either the diel treatment or the light or dark treatment (p<0.05).

**Figure S4:** Sequence-structure based analyses of the ESFC-1 CMBase. **(A)** StralCP dendrogram and clusters for proteins identified in PDB with predicted structural similarity to A2994. Proteins with known enzyme activity have the EC number listed together with corresponding PDB chain ID labels. **(B)** List of residue-residue correspondences between the structural model of A2994 and clcD from *Pseudomonas knackmussii* (PDB chain 4u2b_A; resolution, 1.70 Angstroms; sequence identity, 26%) in critical functional positions. Catalytic triad residues are colored in red. Mutation positions that improve activity toward substrates are in green. In blue are colored positions where mutations increase substrate accessibility during catalysis by increasing flexibility of the loop containing catalytic triad histidine. The distances between corresponding *C-alpha* atoms from calculated structure superposition are shown in the last column. Positions marked by ‘*’ show the perfect agreement between residues from A2994 and native or mutated residues from 4u2bA. **(C)** Cartoon representation of the structural superposition of A2994 model (grey) with the crystal structure of clcD dienelactone hydrolase (yellow). Side chain conformations of corresponding residues identified as functionally critical are shown in “ball and stick” form using the same coloring scheme as in table (B) and numbering from A2994.

**Figure S5:** Cyanobacteria total (intracellular and extracellular) proteome differences between light and dark treatments after 3 days. Total proteome relative abundances were determined via iTraq isobaric tags, and points represent log_2_ normalized median-centered averages of five biological replicates. Colored points are proteins with significant differences in average abundance (>2 standard deviations from mean difference). ‘pfkA’ is 6-phosphofructokinase, ‘ABC.2A’ is an ABC-type multidrug transport system, ‘glnB’ is nitrogen regulatory protein P-II, ‘AfuA’ is an ABC-type Fe3+ transport system, ‘CphX’ is a CO2 hydration protein, ‘phnD’ is an ABC-type phosphate/phosphonate transport system, ‘petJ’ is cytochrome c6, and ‘ABC.PA’ is an ABC-type polar amino acid transport system.

**Supplemental Table Legends**

**Table S1**: Number of cells analyzed via NanoSIMS

**Table S2**: O_2_ and pH measurements

**Table S3:** Proteomics results. Exoproteins and total proteins identified in cyanobacteria and heterotrophs under all experimental conditions. Worksheets include:

**Exo_Dark_up:** Exoproteins significantly overrepresented in the continuous dark treatment, relative to either the diel or continuous light treatment.

**Exo_Light_up:** Exoproteins significantly overrepresented in the continuous light treatment, relative to either the diel or continuous dark treatment.

**Exo_Dark:** Exoproteins significantly overrepresented in the ‘EPS’ fraction relative to the ‘total’ fraction in the continuous dark treatment.

**Exo_Light:** Exoproteins significantly overrepresented in the ‘EPS’ fraction relative to the ‘total’ fraction in the continuous light treatment.

**Exo_Diel:** Exoproteins significantly overrepresented in the ‘EPS’ fraction relative to the ‘total’ fraction in the continuous light treatment.

**Exo_All:** All proteins detected in the ‘EPS’ fractions. Abundance displayed for proteins detected in at least 3 biological replicates and one technical replicate (4-10 replicates total).

**Total_Summary:** Summary of proteins detected in the ‘total’ (intracellular and extracellular) proteomics experiment assigned to each taxonomic group and significantly overrepresented in the continuous light or continuous dark treatment. Taxonomic groups are all 6 representative genomes used in custom search database. ‘% of total OTUs’ are relative abundance estimates based on16S sequencing results. Red indicates taxonomic groups in which the number of proteins identified was lower than predicted based on relative abundance.

**Total_ESFC1:** All proteins detected from the ESFC-1 genome in the ‘total’ proteomics experiment.

**Total_Gamma_Marinobacter_ES048:** All proteins detected from the ES.048 genome in the ‘total’ proteomics experiment.

**Total_Alpha_Hphoto:** All proteins detected from the *H.phototrophica* genome in the ‘total’ proteomics experiment.

**Total_other taxa:** All proteins detected from the ES.050, Tropicibacter multivorans DSM 26470, Algoriphagus terrigena DSM 22685 and the Tepidiphilus thermophilus JCM 19170 genomes in the ‘total’ proteomics experiment.

**Table S4**: Relative abundances of bacterial taxa based on 16S iTag sequencing in light and dark treated biofilms.
