# Supplementary figures and images for: Light Regimes Shape Utilization of Extracellular Organic C and N in a Cyanobacterial Biofilm
Source: mBio. 2016 Jun 28;7(3):e00650-16. doi: 10.1128/mBio.00650-16 (PMC4937211; doi:10.1128/mBio.00650-16)

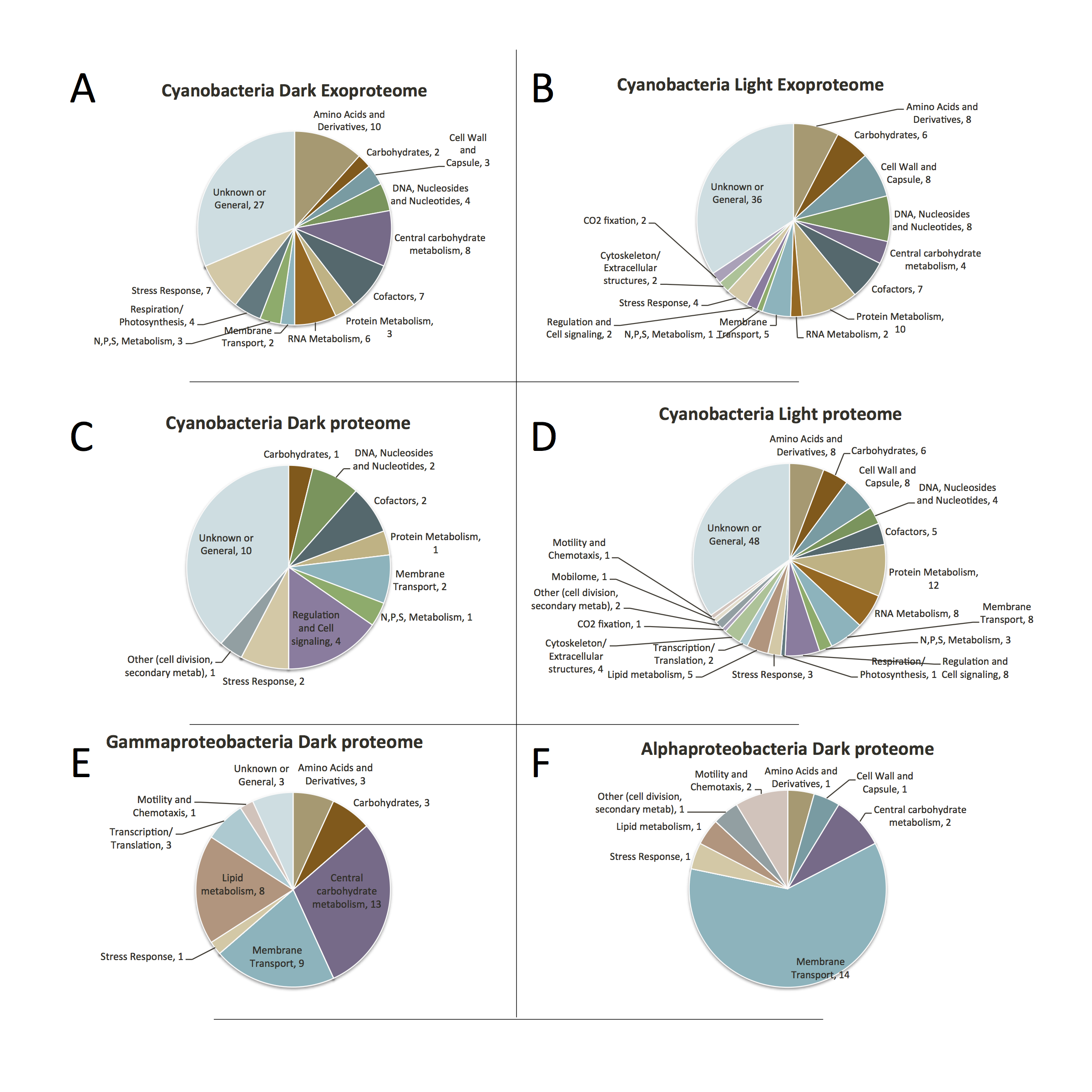

Supplement: Figure S3 — Functional predictions for proteins significantly overrepresented in either continuous dark (panels A and C and panel E) or continuous light (panels B and D and panel F) treatments. (A and B) The top panels show functional categories for ESFC-1 exoproteins overrepresented in either continuous dark (A) or continuous light (B) treatments. (C and D) Middle panels show functional categories for ESFC-1 total proteome proteins overrepresented in either continuous dark (C) or continuous light (D) treatments. (E and F) The bottom panels show functional categories for total proteome heterotroph proteins overrepresented in the continuous dark treatment from representative gammaproteobacterium Marinobacter sp. strain ES.048 (E) or representative alphaproteobacterium H. phototrophica (F). Significantly overrepresented, the abundance of the proteins was higher that seen with either the diel treatment or the light or dark treatment (P < 0.05). Download [file mbo003162866sf3.tif]
