# Supplementary material for: Light Regimes Shape Utilization of Extracellular Organic C and N in a Cyanobacterial Biofilm
Source: mBio. 2016 Jun 28;7(3):e00650-16. doi: 10.1128/mBio.00650-16 (PMC4937211; doi:10.1128/mBio.00650-16)
Supplement: Table S1 — Number of cells analyzed via NanoSIMS. [file mbo003162866st1.docx]

**Table S1**: Number of cells analyzed via NanoSIMS

| Treatment | ESFC-1 | Heterotrophs |
| --- | --- | --- |
| Diel 2h Rep. 1 | 7 | 8 |
| Diel 2h Rep. 2 | 7 | 2 |
| Diel 6h Rep. 1 | 5 | 2 |
| Diel 6h Rep. 2 | 4 | 0 |
| Diel 12h Rep. 1 | 8 | 11 |
| Diel 12h Rep. 2 | 8 | 6 |
| Diel 24h Rep. 1 | n/a | n/a |
| Diel 24h Rep. 2 | 25 | 31 |
| Dark 2h Rep. 1 | 15 | 5 |
| Dark 2h Rep. 2 | 10 | 3 |
| Dark 6h Rep. 1 | 12 | 6 |
| Dark 6h Rep. 2 | 9 | 0 |
| Dark 12h Rep. 1 | 5 | 15 |
| Dark 12h Rep. 2 | 12 | 17 |
| Dark 24h Rep.1 | 11 | 15 |
| Dark 24h Rep. 2 | 21 | 20 |
| Light 24h Rep. 1 | 8 | 8 |
| Light 24h Rep. 2 | 10 | 34 |
| DCMU 6h Rep. 1 | 13 | 36 |
| DCMU 6h Rep. 2 | 11 | 35 |
| Killed Rep. 1 | 5 | 2 |
| Killed Rep. 2 | 10 | 3 |
