# Supplementary material for: Light Regimes Shape Utilization of Extracellular Organic C and N in a Cyanobacterial Biofilm
Source: mBio. 2016 Jun 28;7(3):e00650-16. doi: 10.1128/mBio.00650-16 (PMC4937211; doi:10.1128/mBio.00650-16)
Supplement: Table S2 — O2 and pH measurements. [file mbo003162866st2.docx]

**Table S3**: O_2_ and pH measurements

| Treatment | Average O_2_ at surface (% of air) | pH |
| --- | --- | --- |
| Diel | 189.84 (±19.74) | 9.12 (±0.11) |
| Light | 143.39 (±18.66)* | 9.92 (±0.40)* |
| Dark | 105.62 (±2.63)* | 8.25 (±0.11)* |

* indicates significant difference in mean value compared to diel treatment (p<0.05)
