# Supplementary material for: Light Regimes Shape Utilization of Extracellular Organic C and N in a Cyanobacterial Biofilm
Source: mBio. 2016 Jun 28;7(3):e00650-16. doi: 10.1128/mBio.00650-16 (PMC4937211; doi:10.1128/mBio.00650-16)
Supplement: Table S4 — Relative abundances of bacterial taxa based on 16S iTag sequencing in biofilms treated in the light and the dark. [file mbo003162866st4.docx]

**Table S5**: Relative abundances of bacterial taxa based on 16S iTag sequencing in light and dark treated biofilms

| OTU ID | Dark %^a^ | Dark sd | Light %^a^ | Light sd | Phylum | Class | Order | Family | Genus |
| --- | --- | --- | --- | --- | --- | --- | --- | --- | --- |
| 1 | 35.48 | 7.74 | 56.98 | 5.66 | Cyanobacteria | Oscillatoriophycideae | Oscillatoriales | Phormidiaceae | Microcoleus |
| 2 | 17.91 | 2.73 | 7.06 | 2.63 | Bacteroidetes | Flavobacteriia | Flavobacteriales |  |  |
| 3 | 2.27 | 1.03 | 0.32 | 0.17 | Bacteroidetes | Flavobacteriia | Flavobacteriales | Flavobacteriaceae |  |
| 4 | 0.89 | 0.18 | 0.81 | 0.01 | Bacteroidetes | Flavobacteriia | Flavobacteriales | Flavobacteriaceae | Muricauda |
| 5 | 2.03 | 0.29 | 0.29 | 0.08 | Proteobacteria | Gammaproteobacteria | Alteromonadales | Alteromonadaceae | Glaciecola |
| 6 | 17.14 | 1.23 | 8.54 | 0.91 | Proteobacteria | Gammaproteobacteria | Alteromonadales | Alteromonadaceae | Marinobacter |
| 7 | 0.52 | 0.17 | 1.14 | 0.65 | Proteobacteria | Alphaproteobacteria |  |  |  |
| 8 | 1.04 | 0.18 | 1.64 | 0.47 | Proteobacteria | Alphaproteobacteria | Kiloniellales |  |  |
| 9 | 1.76 | 0.46 | 0.97 | 0.31 | Proteobacteria | Alphaproteobacteria | Rhizobiales | Phyllobacteriaceae |  |
| 10 | 3.74 | 0.23 | 2.50 | 2.87 | Proteobacteria | Alphaproteobacteria | Rhizobiales | Phyllobacteriaceae |  |
| 11 | 1.00 | 0.10 | 1.26 | 0.37 | Proteobacteria | Alphaproteobacteria | Rhizobiales | Phyllobacteriaceae |  |
| 12 | 1.46 | 0.05 | 2.88 | 0.29 | Proteobacteria | Alphaproteobacteria | Rhizobiales | Hyphomicrobiaceae |  |
| 13 | 2.41 | 0.53 | 2.05 | 0.15 | Proteobacteria | Alphaproteobacteria | Rhodobacterales | Hyphomonadaceae | Oceanicaulis |
| 14 | 0.85 | 0.54 | 0.97 | 0.19 | Proteobacteria | Alphaproteobacteria | Rhodobacterales | Rhodobacteraceae |  |
| 15 | 2.61 | 0.17 | 2.04 | 0.65 | Proteobacteria | Alphaproteobacteria | Rhodospirillales | Rhodospirillaceae |  |
| 16 | 1.26 | 0.54 | 1.15 | 0.11 | Proteobacteria | Alphaproteobacteria | Sphingomonadales | Erythrobacteraceae | Erythrobacter |
| 17 | 2.61 | 1.38 | 3.46 | 0.65 | Bacteroidetes | Cytophagia | Cytophagales | Cyclobacteriaceae |  |
| 18 | 2.05 | 0.78 | 2.51 | 0.09 | Planctomycetes | Phycisphaerae | Phycisphaerales |  |  |

^a^ % counts of total rarefied counts, mean of two biological replicates
